# Socio-Demographic, Self-Control, Bullying, Parenting, and Sleep as Proximal Factors Associated with Food Addiction among Adolescents

**DOI:** 10.3390/bs12120488

**Published:** 2022-12-01

**Authors:** Mark Leary, Kirrilly M. Pursey, Antonio Verdejo-Garcia, Scarlett Smout, Nyanda McBride, Bridie Osman, Katrina E. Champion, Lauren A. Gardner, Hiba Jebeile, Erin V. Kelly, Louise Thornton, Maree Teesson, Tracy L. Burrows

**Affiliations:** 1 School of Health Sciences, College of Medicine, Health and Wellbeing, University of Newcastle, Callaghan, Newcastle, NSW 2308, Australia; 2 Hunter Medical Research Institute, New Lambton Heights, Newcastle, NSW 2305, Australia; 3 Priority Research Centre for Physical Activity and Nutrition, University of Newcastle, Callaghan, Newcastle, NSW 2308, Australia; 4 School of Psychological Sciences, Turner Institute for Brain and Mental Health, Monash University, Clayton, VIC 3800, Australia; 5 The Matilda Centre for Research in Mental Health and Substance Use, The University of Sydney, Sydney, NSW 2006, Australia; 6 National Drug Research Institute, EnAble Institute, Curtin University, Perth, WA 6845, Australia; 7 Children’s Hospital Westmead Clinical School, The University of Sydney, Sydney, NSW 2006, Australia; 8 School of Public Health and Community Medicine, University of New South Wales, Kensington, Sydney, NSW 2052, Australia

**Keywords:** food addiction, adolescence, YFAS-C, children’s yale food addiction scale, self-control, Health4Life

## Abstract

Adolescence is considered an important period of neurodevelopment. It is a time for the emergence of psychosocial vulnerabilities, including symptoms of depression, eating disorders, and increased engagement in unhealthy eating behaviours. Food addiction (FA) in adolescents is an area of study where there has been substantial growth. However, to date, limited studies have considered what demographic characteristics of adolescents may predispose them to endorse greater symptoms of FA. Studies have found a variety of factors that often cluster with and may influence an adolescent’s eating behaviour such as sleep, level of self-control, and parenting practices, as well as bullying. Therefore, this study investigated a range of socio-demographic, trait, mental health, and lifestyle-related profiles (including self-control, parenting, bullying, and sleep) as proximal factors associated with symptoms of FA, as assessed via the Yale Food Addiction Scale for Children (YFAS-C) in a large sample of Australian adolescents. Following data cleaning, the final analysed sample included 6587 students (age 12.9 years ± 0.39; range 10.9–14.9 years), with 50.05% identifying as male (*n* = 3297), 48.5% as female (*n* = 3195), 1.02% prefer not to say (*n* = 67), and 0.43% as non-binary (*n* = 28). Self-control was found to be the most significant predictor of total FA symptom score, followed by female gender, sleep quality, and being a victim of bullying. Universal prevention programs should therefore aim to address these factors to help reduce the prevalence or severity of FA symptoms within early adolescent populations.

## 1. Introduction

Adolescence (10–19 years) is a period of neurodevelopment in which the brain, emotions, cognition, and behaviour can be shaped in response to the context in which one is exposed [1,2]. It is a stage of life where significant biological, psychological, and social transformations occur [3], usually characterized by an immature impulse control and low risk perception, often leading to poor self-regulation (which may predict psychological well-being within this time period) [4,5,6]. It is a period of intense maturation and behavioural changes and considered a critical window for the emergence of psychosocial vulnerabilities, including symptoms of depression [3,7], eating disorders [8,9], and increased engagement in unhealthy eating behaviours such as the consumption of takeaway foods, snacking between meals, grazing behaviours, and eating away from home [10,11].

Adolescence is also a time where the development of weight-related issues [5,12] and associated risk factors such as body image dissatisfaction, weight, or mental health concerns including low mood and/or depression [13] emerge and have been shown to carry into adulthood. Furthermore, adolescent obesity rates are considered to be high worldwide [4] and there has been an increase in existing publications investigating disordered eating behaviours, namely overeating under the scope of food addiction (FA). Currently, there is not a universally accepted definition of what FA is, however the term generally refers to the excessive and uncontrolled consumption of highly palatable foods within an addictive-like pattern of eating, commonly operationalised through the Yale Food Addiction Scale (YFAS) [4]. It has been suggested that highly palatable and ultra-processed foods in combination with individual differences and environmental influences may uniquely activate the reward system of the brain, triggering and reinforcing eating behaviours such as addictive eating or FA in susceptible individuals [14,15].

Other factors that often cluster with and may influence an adolescent’s eating behaviour include sleep [16], level of self-control [17], and parenting practices [18], as well as bullying [19]. Studies have found links between sleep duration (short: <4–5 h per night; and/or long: >9 h per night) and higher weight status and unhealthy eating behaviours, including higher intakes of energy and fat as well as excessive calorie consumption from snacks [16,20,21]. Similarly, an individual’s level of self-control may also impact various eating behaviours, with one study reporting higher self-control may be associated with less binge eating and drinking [22]. While in a recent study it has been suggested that trait self-control may lead to a reduced desire for unhealthy foods [23].

Child and adolescent research indicates that parenting practices such as parental monitoring (an awareness of children’s whereabouts and knowledge of their activities) [24] and parental control are associated with eating behaviours, mental health, and behavioural issues. International studies involving adolescent school populations have reported that higher parental monitoring was associated with lower adolescent depressive symptoms over time [25] and that a lack of parental monitoring or control was associated more strongly with delinquent and anti-social behaviours [26,27]. With respect to food, there are limited studies that have examined the association between parental monitoring and adolescent eating behaviours, as this research is often conducted in younger child populations [28]. Within the context of parental control of feeding practices, especially restrictive feeding practices, there are some associations with overeating and poorer self-regulation of energy intake [29]. Lastly, studies that have investigated school bullying victimisation, which has been shown to range between 20% and 45% [30], have found associations with post-traumatic stress symptoms and eating behaviours, such as the overconsumption of highly palatable foods, where the victims of trauma may find themselves coping via the stress-induced consumption of fast food and soda drinks [31,32].

A complex range of factors interplay and influence eating behaviours as outlined above, with a large proportion of the existing research in younger children [13,33]. It is unknown to what extent, if any, these factors contribute to the development of FA specifically in adolescents. Food addiction in adolescents is an area of study where there has been substantial growth in recent years, however prevalence data are currently limited in an Australian context [34]. Two recent international systematic reviews found the prevalence of FA to be estimated at 15% for all participants (12% for community samples and 19% for youth with overweight/obesity) [35] as well as ranging from 2.6% to 49.9% in non-clinical and clinical populations, respectively [9]. Previous reviews of FA demonstrate associations with depressive and anxiety symptoms and eating disorders [9,34], higher weight status [35], and, more recently, higher prevalence in those of lower socio-economic status with a higher body mass index (BMI) [36]. In adolescence, it has been reported that the severity of FA is more likely to be in the mild—moderate range compared to adult populations who are more likely to be in the severe category [9]. Most studies to date have been predominantly conducted with female samples in the European context, with limited studies considering the demographic characteristics that may predispose adolescents to FA [9]. Therefore, it is timely to assess the relationships between FA symptoms and a range of factors in adolescents, particularly within the Australian context.

Altogether, since adolescence is a critical period of neurodevelopment and vulnerability, early identification and treatment of adolescents at risk of experiencing FA may help to prevent the severity or reduce the long-term impact of addictive-like eating behaviours and associated complications. If FA within an adolescent context follows a similar trajectory as substance addiction, obesity, and eating disorders, then adolescents exhibiting addictive-like eating behaviours may be at greater risk for FA and subsequent physical and mental health consequences that persists into adulthood [12]. The adolescent period may, therefore, provide an opportunity for early intervention before the addictive eating behaviours become entrenched. This study will therefore investigate socio-demographic, trait, mental health, and lifestyle-related profiles (including self-control, parenting, bullying, and sleep) as proximal factors associated with symptoms of FA in a large sample of Australian adolescents aged 11–15 years.

## 2. Materials and Methods

### 2.1. Participants and Procedure

The current study is a secondary analysis of data from 6640 participants that was collected at baseline from July to November 2019 as part of the Health4Life trial [37]. The Health4Life trial is a cluster randomised control trial conducted among year 7 students (aged 11–14 years) in 71 independent (*n* = 38), Catholic (*n* = 9), and government (*n* = 24) secondary schools in New South Wales (*n* = 37), Western Australia (*n* = 16), and Queensland (*n* = 18), Australia. The trial aims to evaluate an eHealth prevention program designed to target six lifestyle risk behaviours including: alcohol use, smoking, poor diet, physical inactivity, sleep, and sedentary recreational screen time [37]. Students completed an online self-report questionnaire in a supervised classroom setting [37]. Ethics approval was provided by the Human Research Ethics Committees of the University of Sydney (2018/882), the University of Queensland (2019000037), Curtin University (HRE2019-0083), the NSW Department of Education (SERAP no. 2019006), and the relevant ethics committees of each participating school. Further details, including sample size calculations, recruitment procedures, and consent procedures can be found in the published study protocol [37].

### 2.2. Measures

#### 2.2.1. Demographic Characteristics

Demographic variables included age, gender, self-reported height, and weight used to calculate BMI, postcode, and family affluence. Postcode data were coded post data collection for socio-economic status via the Socio-Economic Indexes for Areas (SEIFA) scale [38]. The BMI z-score was calculated post data collection with cut off points categorised based on the World Health Organization guidelines [39]. The BMI z-score categories (overweight and obesity) were based on the United States fitted Lambda Mu Sigma (LMS) curves [40].

#### 2.2.2. Parental Control and Parental Monitoring

Parental control was assessed by a modified 7-item version of the validated Parenting Strategies for Eating and Activity Scale (PEAS) [41], where respondents were asked the degree to which their parents kept track of certain dietary and physical activity behaviours such as “how much do your parents keep track of the high-fat foods you eat?”. The tool includes responses on a 5-point Likert-type scale (from 1—“never” to 5—“always”) scored out of 35, where higher scores indicate greater parental control. Parental monitoring was assessed through the Small Parenting Monitoring Scale (SPMS) [42] where respondents were asked the degree to which their parents have knowledge of their activities such as “my parent(s) usually know what I am doing after school” and “I tell my parent(s) who I am going to be with before I go out”. The SPMS uses a 5-point Likert-type scale (from 0—“never” to 4—“always”) scored out 24, where higher scores indicate greater parental monitoring.

#### 2.2.3. Family Affluence

Relative family affluence was measured using the validated six-item Family Affluence Scale III (FASIII) [43], which generates an individual affluence score out of 13 based on a set of indicators of wealth such as “how many computers does your family own?”. Higher scores indicate greater family affluence.

#### 2.2.4. Addictive Eating

Addictive eating behaviours were assessed by the validated Children’s Yale Food Addiction Scale (YFAS-C) [44] that consists of 25-items, in dichotomous and Likert-type format (from 0—“never” to 4—“always”). The YFAS-C provides a FA symptom score based on similar criteria for substance use disorder of the Diagnostic and Statistical Manual of Mental Disorders IV (DSM-IV). Respondents were asked to think of specific foods they have difficulty controlling the consumption of within the past 12 months (e.g., chocolate, pizza, chips, and soft drinks) and select how frequently they experienced a range of situations, for example: “when I start eating, I find it hard to stop” or “I avoid places where I cannot eat the food I want”. Symptom scores can range from zero to seven and include: (1) substance taken in larger amounts, (2) persistent desire/unsuccessful attempts to quit, (3) substantial time spent to obtain/use/recover, (4) giving up important social/occupational/recreational activities, (5) continued use despite adverse consequences, (6) tolerance, and (7) withdrawal. Additionally, the YFAS-C also assesses clinically significant impairment or distress from eating. Individuals who endorsed three or more symptoms plus the “significant impairment” criteria met the diagnosis of “food addiction”. However, for the purpose of this study, a “food addiction symptom score” will be the focus as opposed to a diagnosis as this has been shown to be more appropriate and sensitive in a non-clinical adolescent sample [44].

#### 2.2.5. Self-Control

Self-control was assessed by the validated Brief Self-Control Scale [22], which is a 13-item questionnaire that measures levels of trait self-control such as “I am good at resisting temptation” and “I wish I had more self-discipline”. The survey uses a 5-point Likert-type scale (from 1—“not at all” to 5—“very much”) and is scored between 13 and 65 where higher scores indicate greater trait self-control.

#### 2.2.6. Bullying

The degree of bullying perpetration or being a victim of bullying at school was assessed through a shortened version of the original 36-item Olweus Bully/Victim Questionnaire [45]. Students were asked two dichotomous items “have you ever been bullied?” and “have you ever bullied others?”. If respondents answered “yes” to either of these items, they were asked to indicate how often they were bullied/bullied others within the past year via a 6-point Likert-type format (from 0—"never” to 5—“more than once a week”). These two dichotomous questions have not been validated.

#### 2.2.7. Sleep

Sleep was assessed via three tools including: the Paediatric Daytime Sleepiness Scale (PDSS) [46], modified Sleep Habits Survey (SHS) [47], and a bespoke question developed specifically for the Health4Life study capturing difficulty falling asleep. The PDSS is an 8-item questionnaire designed to assess daytime sleepiness where respondents were asked the degree to which they feel sleepy throughout the day such as “how often do you fall asleep or feel drowsy in class?” and “how often do you fall back to sleep after being woken in the morning?”. It uses a 5-point Likert-type scale (from 0—"never” to 4—“very often/always”) and scored out of 32 where higher scores indicate greater sleepiness. The survey was also scored dichotomously as “excessive” versus “not excessive daytime sleepiness”. The modified SHS survey contains 6-items relating to bedtime, wake time, and total sleep time for both school nights and weekends during the previous week and asks respondents to answer questions such as “what time did you usually go to bed?” and “how long did it take you to fall asleep?”. Respondents were required to record the times for each item in hours and minutes. Based on their times, respondents were then categorised to experience “normal sleep”, “under sleep”, or “over sleep” as well as “at risk” or “not at risk” for negative health outcomes based on national guidelines. Respondents for the developed bespoke question “do you have difficulty falling asleep?” were required to indicate the level of difficulty falling asleep on a 5-point Likert type scale (from 0—“no difficulty” to 4—“very severe”).

### 2.3. Data Analysis

To minimise missing data, analysis was completed on the prorated data using STATA version 16.1 [48]. A total of 53 participants were excluded from the data analysis based on the following criteria: (1) age was older than 15 years, (2) BMI z-score lower than −4.0 or higher than +5.0, and (3) non-plausible responses based on the “different identity” gender category. The World Health Organization (WHO) guidelines of fixed exclusion range BMI z-scores lower than −4.0 or higher than +5.0 are considered to be biologically implausible values and excluded from the analysis. Typically, these values (outliers) are the result of misreporting, rather than from true growth extremes [39]. A descriptive analysis, including mean ± SD, was used to explore participant characteristics by gender (age, BMI z-score, socio-economic status, family affluence, self-control, parenting, sleep, and total FA (YFAS-C) symptoms). To examine the relationship between total FA (YFAS-C) symptom scores and categorical outcome variables (gender, family affluence, sleep, BMI z-score, and bullying), we used Analysis of Variance (ANOVA) tests and *t*-tests. To determine the relationship between total FA (YFAS-C) symptom scores and outcome variables (gender, socio-economic status, BMI z-score, sleep, parenting, family affluence, bullying, and self-control), we used multivariate linear regression models.

### 2.4. Missing Data Analysis

Due to a substantial amount of missing data for the YFAS-C and SEIFA (>15%), a missing data analysis was performed of both outcomes on the four socio-demographic variables (gender, age, BMI z-score, and socio-economic status) via the Pearson chi^2^ test (Table 1). A significant difference in the missing and captured data for the YFAS-C and SEIFA was found across both gender (*p* = 0.001 and *p* = 0.001) and age (*p* = 0.001 and *p* = 0.003) categories. Overall, males and those participants within the 13-year age group had the most missing YFAS-C and SEIFA data. Of those participants that recorded their BMI (*n* = 2179), 12.5% (*n* = 272), and 25.5% (*n* = 555) were missing YFAS-C and SEIFA data, respectively. Of those participants that reported a valid postcode and subsequently issued with a SEIFA number (*n* = 4115), 12.3% (*n* = 508) were missing YFAS-C data. Of those participants that received a YFAS-C symptom score (*n* = 5649), 36.1% (*n* = 2042) were missing SEIFA data.

## 3. Results

### 3.1. Participants Characteristics

Following data cleaning, the final analysed sample included 6587 students (age 12.9 years ± 0.39; range 10.9–14.9 years), with 50.05% identifying as male (*n* = 3297), 48.5% as female (*n* = 3195), 1.02% prefer not to say (*n* = 67), and 0.43% as non-binary (*n* = 28). The average BMI z-score was −0.19 ± 1.13 placing them in the “healthy weight” category (range −3.99 to 2.86), with an average BMI z-score of −0.10 ± 1.15 for males (*n* = 1247), −0.31 ± 1.09 for females (*n* = 926), 0.78 ± 0.74 for prefer not to say (*n* = 4), and 0.95 ± 1.27 for non-binary (*n* = 2). The number of males categorised as “overweight” and “obesity” were 213 (9.8%) and 32 (1.5%), respectively. The number of females categorised as “overweight” and “obesity” were 88 (4.0%) and 8 (0.4%), respectively. The number of prefer not to say categorised as “overweight” and “obesity” were 1 (0.01%) and zero (0%), respectively. The number of non-binary categorised as “overweight” and “obesity” were zero (0%) and 1 (0.01%), respectively. The average socio-economic status (SEIFA) and family affluence score was 6.99 ± 2.64 (*n* = 4115) and 9.34 ± 1.92 (*n* = 5979), respectively, with females reporting the highest average SEIFA score of 7.24 ± 2.60 (48.8%) and highest average family affluence score of 9.51 ± 1.84 (49.4%). Males reported the lowest average SEIFA score of 6.75 ± 2.67 (50.2%) and non-binary reported the lowest average family affluence score of 8.32 ± 2.30 (0.4%). The average self-control score was 44.91 ± 8.06 (*n* = 5732), with females reporting the highest average score of 45.77 ± 7.98 (49.5%) and non-binary reporting the lowest average score of 38.64 ± 8.37 (0.4%). The average scores of both parental monitoring and parental control were 20.98 ± 4.22 (*n* = 6121) and 26.13 ± 6.24 (*n* = 6094), respectively, with females reporting the highest average scores for both of 21.89 ± 3.36 (49.3%) for parental monitoring and 26.80 ± 6.08 (49.4%) for parental control. Non-binary reported the lowest average score for both parental monitoring and parental control of 18.5 ± 4.57 (0.4%) and 23.04 ± 6.47 (0.4%). The average paediatric daytime sleepiness scale score was 13.88 ± 6.12 (*n* = 6556), with prefer not to say reporting the highest average score of 17.15 ± 6.24 (1.0%) and males reporting the lowest average score of 13.25 ± 6.06 (50.0%). Table 2 displays the socio-demographic characteristics and FA (YFAS-C) scores of the sample organised by self-reported gender identity.

### 3.2. Addictive Eating Symptoms by Gender

Of the seven possible FA (YFAS-C) symptoms, the mean number of symptoms met was 1.35 ± 1.45 (males 1.31 ± 1.38, females 1.37 ± 1.52, prefer not to say 1.85 ± 1.60, and non-binary 1.8 ± 1.44, *p* = 0.011). Overall, 82% (*n* = 4634) of the participants endorsed less than three symptoms (males *n* = 2300, females *n* = 2278, prefer not to say *n* = 37 and non-binary *n* = 19) and 18% (*n* = 1015) endorsed three or more symptoms (males *n* = 473, females *n* = 518, prefer not to say *n* = 18 and non-binary *n* = 6).

The most frequently endorsed symptom was symptom 2, “persistent desire/unsuccessful attempts to quit” (*n* = 2275 at 40.3%) and symptom 4 “important social activities given up” (*n* = 1656 at 29.3%) (Table 3).

### 3.3. Association between Categorical Indicators of Social, Lifestyle, and Mental Health Status and Addictive Eating

As per Table 4, all categorical variables showed a significant relationship to total FA (YFAS-C) symptom scores, except for male BMI z-scores. Those students that did not report gender or identified as non-binary, reported higher total FA (YFAS-C) symptom scores (“prefer not to say” 1.85 ± 1.6 and “non-binary” 1.8 ± 1.44, compared to 1.31 ± 1.38 for males and 1.37 ± 1.52 for females). The Females in the “overweight” and “obesity” categories for BMI z-score were found to have higher total FA (YFAS-C) symptom scores compared to the “healthy weight” category (1.88 ± 1.78 and 3.0 ± 2.53, compared to 1.31 ± 1.47, *p* < 0.001). Although not statistically significant, males in the “overweight” and “obesity” categories for BMI z-score were also found to have higher total FA (YFAS-C) symptom scores compared to the “healthy weight” category (1.36 ± 1.46 and 1.76 ± 1.88, compared to 1.22 ± 1.27, *p* = 0.052). On average, the greater the difficulty getting to sleep or experiencing daytime sleepiness was associated with higher total FA (YFAS-C) symptom scores (3.06 ± 2.20 “very severe” and 1.74 ± 1.66 “excessive daytime sleepiness” compared to 1.15 ± 1.30 “no difficulty” and 1.04 ± 1.19 “not excessive daytime sleepiness”, *p* < 0.001). Over-sleeping or under sleeping were both associated with higher total FA (YFAS-C) symptom scores compared to normal sleep (1.50 ± 1.42, 1.48 ± 1.53 and 1.18 ± 1.35, respectively, *p* < 0.001). Being deemed “at risk” based on not meeting National sleep guidelines was associated with higher total FA (YFAS-C) symptom scores compared to “not at risk” (1.49 ± 1.52 versus 1.18 ± 1.35, *p* < 0.001). The greater the frequency of reported victims of bullying or bullying others was also associated with the endorsement of greater total FA (YFAS-C) symptoms (2.22 ± 2.08 “more than once a week” compared to 1.28 ± 1.41 never, *p* < 0.001).

### 3.4. All-Inclusive Model of Dimensional Predictors of Addictive Eating

A multiple linear regression was calculated to predict total FA (YFAS-C) symptom scores based on the different outcome variables. The regression model was significant (F(11, 1360) = 26.96, *p* < 0.001) and explained 17% of the variance in FA. The outcome variable “self-control” was the most significant predictor of the total FA (YFAS-C) symptom score (−0.053 [95% CI: −0.064 to −0.043], *p* < 0.001) followed by “female gender” (0.215 [95% CI: 0.066 to 0.363], *p* = 0.005), “sleep quality (PDSS)” (0.017 [95% CI: 0.004 to 0.031], *p* = 0.013), and “victim of bullying” (0.055 [95% CI: 0.002 to 0.108], *p* = 0.040) (Table 5).

Although not indicated as significant in the regression model (Table 5), both socio-economic status (SEIFA) and BMI z-scores were significantly correlated with total FA (YFAS-C) scores in unadjusted models. There was a negative correlation between SEIFA and total FA (YFAS-C) symptom scores overall, indicating that SEIFA scores decreased as YFAS-C symptom scores increased (r = −0.045, *p* = 0.009, (*n* = 3572). This correlation was not found to be significant for males (r = −0.024, *p* = 0.32 (*n* = 1779), but was found to be significant for females (r = −0.066, *p* = 0.005 (*n* = 1793). There was a positive correlation between the BMI z-score and total FA (YFAS-C) symptom scores overall, indicating that as BMI z-score increased YFAS-C symptom scores also increased (r = 0.063, *p* = 0.006, (*n* = 1901). This correlation was not found to be significant for males (r = 0.026, *p* = 0.39, (*n* = 1073), but was found to be significant for females (r = 0.115, *p* < 0.001, (*n* = 828).

## 4. Discussion

This study sought to explore the relationship between socio-demographic characteristics (age, gender, BMI, and socio-economic status), self-control, parenting, bullying, and sleep as proximal factors of food addiction (FA) symptoms in a large sample of Australian adolescents. Self-control was the most significant predictor of the total FA (YFAS-C) symptom score, followed by being female, poor sleep quality, and being a victim of bullying. The mean number of FA (YFAS-C) symptoms of this group of adolescents was quite low compared to adult populations (1.35 out of a possible 7), with females endorsing slightly higher symptoms compared to males (1.37 versus 1.31). The mean numbers of FA (YFAS-C) symptoms were found to increase with higher difficulty of getting to sleep, excessive daytime sleepiness, under sleeping or over sleeping, increased bullying perpetration/victimization frequency, and higher BMI z-scores.

The mean FA (YFAS-C) symptoms analysed in this sample of adolescents appear to align with similar adolescent studies where FA is most often reported in its mild/moderate form, indicating fewer symptoms are endorsed [9]. In a recent (2021) review of FA in adolescents (*n* = 27 studies) [9], the mean symptom scores ranged from 1.0 to 5.2 with scores found to be higher in clinical samples compared to non-clinical samples. Our study also aligns with this as it is a community-based study, where higher YFAS-C symptom scores may have been expected if participants were recruited from clinical samples. This contrasts to adults where often the overall number of FA symptoms are higher. In a 2014 systematic review of FA prevalence, the weighted mean number of symptoms for adults (*n* = 8 studies) was 2.8 (range from 1.8 to 4.6) out of a possible score of seven, with females reporting higher number of symptoms compared to males [49]. Additionally, more recent reviews of FA within adult populations using the updated YFAS 2.0 report that the total number of symptoms are often in the severe form (≥6 symptoms out of a possible 11) [15,34,50,51]. This contrast between the two age groups may indicate a progression in severity as an individual transitions from adolescence to adulthood. Furthermore, the higher symptom scores observed in females may be due to females being more likely to endorse the clinical impairment or distress criteria of FA diagnosis [52], perceiving that their addictive eating behaviours are more severe compared to males, as well as being more critical of themselves when reporting [53].

Interestingly, this study found that female (but not male) BMI z-scores and socio-economic status (SEIFA) were significantly correlated with the total FA (YFAS-C) symptom score (r = 0.115, *p* < 0.001, and r = −0.066, *p* = 0.005, respectively); however, they were not found to be significant in the regression model. Several studies have recently reported significant positive correlations between FA symptoms and BMI z-scores [54,55,56], indicating that as BMI z-score increases YFAS symptom score also increases. While income appears to be associated with unhealthy eating behaviours and obesity [57], the link between socio-economic status and FA is currently limited with one study reporting similar findings of greater YFAS symptoms related to increased BMI and low household income [36]. Furthermore, students that did not identify as either male or female, reported higher mean FA (YFAS-C) symptom scores (1.85 “prefer not to say” and 1.8 “non-binary”). Some studies that have examined the health of gender diverse adolescents and young adults report a substantially elevated risk for various negative health outcomes compared to cisgender adolescents [58,59,60], including substance use, sexual behaviours, emotional distress, bullying victimization, and eating disorders [61]. Based on the findings of limited studies to date regarding the possible gender differences between associations of BMI z-score and socio-economic status with FA symptom scores, it highlights the importance of reporting across all gender groups where possible, and not just those identifying as males or females. Additionally, future studies may need to assess these variables in more depth to help increase our understanding of these relationships.

The association between FA symptoms and self-control reported in this study is consistent with the findings of recent studies, suggesting that self-control may be a contributing factor to unhealthy eating behaviours, including FA [17,23,62]. A study by Luo et al. (2022), which comprised a cohort of college students, found a significant negative correlation between self-control and FA [63], while other studies of adult populations with FA have reported similar findings of reduced self-control [64,65]. The Brief Self-Control Scale (BSCS), which is widely used for measuring trait self-control in various fields of psychological research [66], is used minimally within an adolescent eating behaviour context. Therefore, previous adolescent studies that have utilised this questionnaire to measure self-control have performed it within the context of body composition measures (i.e., adiposity and leanness) [67,68], where higher levels of trait self-control were associated with lower adiposity (including BMI, waist circumference, and sum of skinfolds) [67]. Given that adolescence is often considered a transitional period [13] and the possible connection between self-control and FA symptom scores, interventions may be best directed at prevention efforts around control within the context of overeating to help reduce the likelihood of developing higher FA symptoms later in adulthood.

Both sleep quality and being a victim of bullying were also found to be significant predictors of total FA (YFAS-C) symptom scores. Specifically, with regard to FA, studies have found that those individuals with FA reported significantly more symptoms of poorer sleep quality compared to non-FA individuals [16,69]. Although research in the area of bullying and FA is limited, the significant predictor of bullying victimisation and total FA (YFAS-C) symptom scores seen in this study aligns with other studies that report associations with bullying and eating disorders. A systematic review and meta-analysis by Øverland Lie et al. (2019) that included 22 studies reported that individuals with eating disorders (bulimia nervosa and binge-eating disorder) were two to threefold more likely than controls to have experienced bullying or teasing prior to eating disorder development [19]. Interventions that focus on improving sleep habits and the prevention of bullying may therefore help reduce the risk of developing FA.

There was little association found between FA (YFAS-C) symptoms with adolescent reported parental monitoring and parental control. Existing studies consider these variables more within the context of youth development, dietary intake, disordered eating, or obesity risk [29,70,71,72]. This current study, therefore, represents an interesting analysis in the context of FA where different findings may be attributed depending on who is the reporter of these behaviours. Baseline values of the parental variables indicate moderately high levels of reported monitoring and control in this sample. Results may have been different if the parenting variables were reported by the parents rather than the adolescents, as parents tend to provide socially desirable answers and avoid reporting problematic behaviours [73]. The lack of association of parental monitoring and FA in this current study is also similar to a previous study (2017) conducted in younger children (5–12 years) where monitoring was assessed with the Child Feeding Questionnaire (CFQ) [33]. Of the three parental feeding practices assessed (restriction, pressure to eat, and monitoring), monitoring was the only variable reported to not have a significant association with FA in children (*p* = 0.29). It was not until the children’s BMI z-score was included in the model that monitoring became significant [33].

Intervention studies that adopt a treatment approach rather than a prevention of FA approach within adolescent populations are currently limited. In a recent (2021) systematic review of the effectiveness of current intervention treatments for FA (*n* = 9 studies), there were two adolescent studies identified [50]. Both studies had small samples (*n* = 35 and *n* = 26, respectively), included overweight/obese adolescents (11–18 years), focused on weight management, and utilised the expertise of various health professionals (e.g., dietitians, psychologists, and physical therapists) [12,74]. While both studies reported an overall reduction in FA diagnosis or symptom score, they were not, however, statistically significant [50]. Given the number of significant predictors of total FA (YFAS-C) symptom score analysed in this study (self-control, gender, sleep quality, and being a victim of bullying), interventions aimed at universal prevention delivered to early adolescents encompassing these factors may be effective at reducing the risk of developing and/or the severity of FA.

The results of this study have several strengths and limitations to consider. To date, the Health4Life trial is the largest study examining FA in an Australian adolescent population [9] with approximately equal numbers of males and females. This contrasts to the adult literature where most studies are carried out with predominantly female populations [49,50], limiting the generalizability. This study explored a number of proximal factors that may increase the risk of adolescents developing FA, which a limited number of studies have performed. Based on this analysis, the proximal factors of self-control, female gender, sleep quality, and being a victim of bullying, which were identified could be used to help inform future prevention programs. Several limitations also need to be considered. While the analysis included participants who did not identify their gender or reported their gender as non-binary, the sample sizes were small. Secondly, the analyses did not account for school level clustering despite data being collected in schools or possible mediating factors, such as youth disposable income or access to fast food outlets, where older students who have more autonomy and more disposable income may be more susceptible to unhealthy eating practices [75]. Thirdly, the data collected in this study were cross-sectional, included a predominately English-speaking cohort, and underrepresented rural/remote areas, which may cause the results to be less generalizable across different ethnic cohorts. Lastly, while there are many advantages of using self-report surveys in research, including low-burden and cost effectiveness [76], some self-report surveys can be prone to bias. Specifically, in this study, the variable of self-control, which was measured by a short self-report survey, may be susceptible to bias as adolescents tend to be highly critical of themselves [77], which may provide a false representation of the levels of self-control reported. A more objectional measure of impulse control such as a Go/no-go task may be more ideal. Additionally, the parenting variable results (parental control and parental monitoring) may have been different if the parents themselves completed the questionnaires rather than the students.

## 5. Conclusions

Secondary analysis of the Health4Life baseline data has highlighted a number of proximal factors that may increase an adolescent’s risk of developing and/or the severity of FA symptoms. These factors include self-control, female gender, sleep quality, and being a victim of bullying. Universal prevention programs delivered to early adolescents should therefore address these factors to help reduce the likelihood of developing higher FA symptoms later in adulthood.

## Figures and Tables

**Table 1:** Missing food addiction (YFAS-C) and Socio-economic Indexes for Areas scale (SEIFA) data reported as frequency (%).

		YFAS-C Missing	YFAS-C Captured	Significance of Difference			SEIFA Missing	SEIFA Captured	Significance of Difference
Gender	Male Female Prefer not to say Non-binary	524 (15.9%) 399 (12.5%) 12 (17.9%) 3 (10.7%)	2773 (84.1%) 2796 (87.5%) 55 (82.1%) 25 (89.3%)	*0.001*	Gender	Male Female Prefer not to say Non-binary	1233 (37.4%) 1185 (37.1%) 41 (61.2%) 13 (46.4%)	2064 (62.6%) 2010 (62.9%) 26 (38.8%) 15 (53.6%)	*0.001*
Age	11 12 13 14	1 (11.1%) 386 (16.4%) 540 (13%) 11 (15.5%)	8 (88.9%) 1974 (83.6%) 3605 (87%) 60 (84.5%)	*0.003*	Age	11 12 13 14	0 (0%) 947 (40.1%) 1494 (36%) 31 (43.7%)	9 (100%) 1413 (59.9%) 2651 (64%) 40 (56.3%)	*0.001*
BMI z-score		272 (12.5%)	1901 (87.5%)		BMI z-score		555 (25.5%)	1618 (74.5%)	
SEIFA		508 (12.3%)	3607 (87.7%)		YFAS-C		2042 (36.1%)	3607 (63.9%)	

BMI, body mass index; YFAS-C, Yale Food Addiction Scale for Children.

**Table 2:** Participant characteristics by gender reported as mean ± S.D, number (*n*) and frequency (%).

	Male	Female	Prefer Not to Say	Non-Binary	Total
Gender	3297 (50.05%)	3195 (48.5%)	67 (1.02%)	28 (0.43%)	6587
Age (years)	12.93 ± 0.39 (Range from 10.95 to 14.98) *n* = 3297 (50.05%)	12.86 ± 0.38 (Range from 11.50 to 14.97) *n =* 3195 (48.5%)	12.96 ± 0.43 (Range from 11.37 to 13.84) *n =* 67 (1.02%)	12.93 ± 0.46 (Range from 12.0 to 14.0) *n =* 28 (0.43%)	12.90 ± 0.39 (Range from 10.95 to 14.98) *n* = 6587
BMI z-score Category *Healthy weight* *Overweight* *Obesity*	−0.10 ± 1.15 (Range from −3.83 to 2.86) *n* = 1247 (57.2%) *n* = 1002 (46%) *n* = 213 (9.8%) *n* = 32 (1.5%)	−0.31 ± 1.09 (Range from −3.99 to 2.70) *n* = 926 (42.5%) *n* = 830 (38.1%) *n* = 88 (4.0%) *n* = 8 (0.4%)	0.78 ± 0.74 (Range from −0.07 to 1.73) *n* = 4 (0.2%) *n* = 3 (0.14%) *n =* 1 (0.01%) *n* = 0 (0%)	0.95 ± 1.27 (Range from 0.05 to 1.85) *n* = 2 (0.1%) *n* = 1 (0.01%) *n* = 0 (0%) *n* = 1 (0.01%)	−0.19 ± 1.13 (Range from −3.99 to 2.86) *n* = 2179 *n =* 1836 *n* = 302 *n =* 41
Socio-Economic Status (SEIFA) (Range from 1 to 10)	6.75 ± 2.67 *n* = 2064 (50.2%)	7.24 ± 2.60 *n* = 2010 (48.8%)	7.15 ± 2.72 *n* = 26 (0.6%)	7.07 ± 2.46 *n* = 15 (0.4%)	6.99 ± 2.64 *n* = 4115
Family affluence scale (Range from 0 to 13)	9.18 ± 1.96 *n* = 2941 (49.2%)	9.51 ± 1.84 *n* = 2952 (49.4%)	8.89 ± 2.25 *n* = 61 (1.02%)	8.32 ± 2.30 *n* = 25 (0.4%)	9.34 ± 1.92 *n* = 5979
Self-control (Range from 13 to 65)	44.19 ± 8.02 *n* = 2813 (49.1%)	45.77 ± 7.98 *n* = 2838 (49.5%)	40.54 ± 8.25 *n* = 56 (1.0%)	38.64 ± 8.37 *n* = 25 (0.4%)	44.91 ± 8.06 *n* = 5732
Parental monitoring (Range from 0 to 24)	20.13 ± 4.73 *n* = 3016 (49.3%)	21.89 ± 3.36 *n =* 3018 (49.3%)	19.51 ± 5.83 *n* = 61 (1.0%)	18.5 ± 4.57 *n* = 26 (0.4%)	20.98 ± 4.22 *n* = 6121
Parental control (Range from 7 to 35)	25.51 ± 6.31 *n =* 2999 (49.2%)	26.80 ± 6.08 *n* = 3008 (49.4%)	25.36 ± 7.25 *n* = 61 (1.0%)	23.04 ± 6.47 *n* = 26 (0.4%)	26.13 ± 6.24 *n* = 6094
Paediatric daytime sleepiness scale (Range from 0 to 32)	13.25 ± 6.06 *n* = 3279 (50.0%)	14.45 ± 6.09 *n* = 3183 (48.6%)	17.15 ± 6.24 *n* = 67 (1.0%)	16.52± 6.94 *n* = 27 (0.4%)	13.88 ± 6.12 *n* = 6556
Total FA Symptoms (Range from 0 to 7)	1.31 ± 1.38 *n =* 2773 (49.1%)	1.37 ± 1.52 *n* = 2796 (49.5%)	1.85 ± 1.60 *n* = 55 (1.0%)	1.8 ± 1.44 *n =* 25 (0.4%)	1.35 ± 1.45 *n* = 5649

BMI, body mass index; FA, food addiction; SEIFA, Socio-economic Indexes for Areas scale (1–10 with 1 being most disadvantaged and 10 being least disadvantaged).

**Table 3:** Yale Food Addiction Scale (YFAS-C) symptom criteria endorsement by gender for the total sample reported as frequency (%).

	Symptom 1	Symptom 2	Symptom 3	Symptom 4	Symptom 5	Symptom 6	Symptom 7	<3 Symptoms	≥3 Symptoms
Male	342 (12.3%)	1086 (39.2%)	182 (6.6%)	854 (30.8%)	253 (9.1%)	536 (19.3%)	386 (13.9%)	2300 (82.9%)	473 (17.1%)
Female	375 (13.4%)	1142 (40.8%)	184 (6.6%)	775 (27.7%)	324 (11.6%)	556 (19.9%)	461 (16.5%)	2278 (81.5%)	518 (18.5%)
Prefer not to say	9 (16.4%)	30 (54.5%)	4 (7.3%)	20 (36.4%)	12 (21.8%)	15 (27.3%)	12 (21.8%)	37 (67.3%)	18 (32.7%)
Non- binary	5 (20%)	17 (68%)	0 (0%)	7 (28%)	3 (12%)	8 (32%)	5 (20%)	19 (76%)	6 (24%)
Total	731 (12.9%)	**2275 (40.3%)**	370 (6.5%)	**1656 (29.3%)**	592 (10.5%)	1115 (19.7%)	864 (15.3%)	4634 (82%)	1015 (18%)

*Symptom 1* = substance taken in larger amount; *Symptom 2* = persistent desire/unsuccessful attempts to quit; *Symptom 3* = much time to obtain; *Symptom 4* = important social activities given up; *Symptom 5* = continued use despite adverse consequences; *Symptom 6* = tolerance; *Symptom 7* = withdrawal.

**Table 4:** Total Food Addiction (YFAS-C) symptom scores by categorical variable for the total sample reported as mean ± S.D and number (*n*).

Variable	Category	YFAS-C Symptom Score ± SD	*p*-Value
Socio-demographic			
Gender	Male	1.31 ± 1.38 (*n* = 2773)	*0.011*
	Female	1.37 ± 1.52 (*n* = 2796)	
	Prefer not to say Non-binary	1.85 ± 1.6 (*n* = 55) 1.8 ± 1.44 (*n* = 25)	
BMI z-score * Male	Healthy weight (<0.91) Overweight (>+0.91) Obesity (>+1.84)	1.22 ± 1.27 (*n* = 862) 1.36 ± 1.46 (*n* = 182) 1.76 ± 1.88 (*n* = 29)	0.052
Female	Healthy weight (<0.97) Overweight (>+0.97) Obesity (>+1.76)	1.31 ± 1.47 (*n* = 746) 1.88 ± 1.78 (*n* = 76) 3.0 ± 2.53 (*n* = 6)	*<0.001*
Sleep			
Sleep Quality (Paediatric daytime sleepiness scale)	Excessive daytime sleepiness Not excessive daytime sleepiness	1.74 ± 1.66 (*n* = 2476) 1.04 ± 1.19 (*n* = 3176)	*<0.001*
Sleep habits (Difficulty getting to sleep)	No difficulty	1.15 ± 1.30 (*n* = 3436)	*<0.001*
	Mild difficulty	1.34 ± 1.47 (*n* = 844)	
	Moderate difficulty	1.68 ± 1.55 (*n* = 933)	
	Severe difficulty	2.03 ± 1.75 (*n* = 350)	
	Very severe difficulty	3.06 ± 2.20 (*n* = 83)	
Sleep score simple (Modified Sleep Habits Survey)	Normal sleep Under sleep Oversleep	1.18 ± 1.35 (*n* = 3090) 1.48 ± 1.53 (*n* = 2047) 1.50 ± 1.42 (*n* = 252)	*<0.001*
Sleep score simple (Modified Sleep Habits Survey)	Not at risk (meeting National sleep guidelines) At risk (not meeting National sleep guidelines)	1.18 ± 1.35 (*n* = 3090) 1.49 ± 1.52 (*n* = 2299)	*<0.001*
Bullying			
Victim of Bullying	Never	1.13 ± 1.29 (*n* = 2516)	*<0.001*
	Not at all	1.25 ± 1.39 (*n* = 1048)	
	Only once or twice	1.48 ± 1.52 (*n* = 1227)	
	From 2 to 3 times a month	1.78 ± 1.66 (*n* = 364)	
	About once a week	2.03 ± 1.68 (*n* = 194)	
	More than once a week	2.01 ± 1.85 (*n* = 268)	
Bullying frequency	Never	1.28 ± 1.41 (*n* = 4893)	*<0.001*
	Not at all	1.64 ± 1.56 (*n* = 193)	
	Only once or twice	1.80 ± 1.63 (*n* = 371)	
	From 2 to 3 times a month	2.30 ± 1.95 (*n* = 56)	
	About once a week	2.20 ± 2.09 (*n* = 20)	
	More than once a week	2.22 ± 2.08 (*n* = 27)	

* BMI z-score categories based on the United States’ cut offs [40] for males and females only, where overweight corresponds to body mass index of 25 kg/m^2^ and obesity corresponds to body mass index of 30 kg/m^2^.

**Table 5:** Multivariate linear regression coefficients of outcome variables and total YFAS-C symptom score.

Variable	β Coef	95% CI	*p*-Value
Gender	0.215	(0.066 to 0.363)	*0.005*
SEIFA	−0.013	(−0.040 to 0.014)	0.340
BMI z−score	0.012	(−0.050 to 0.074)	0.699
Sleep Quality (PDSS)	0.017	(0.004 to 0.031)	*0.013*
Sleep habits (difficulty getting to sleep)	0.076	(−0.001 to 0.153)	0.052
Parental control	−0.010	(−0.022 to 0.002)	0.111
Parental monitoring	−0.006	(−0.026 to 0.013)	0.525
Family affluence scale	−0.033	(−0.072 to 0.006)	0.101
Bullying	0.067	(−0.029 to 0.163)	0.172
Victim of bullying	0.055	(0.002 to 0.108)	*0.040*
Self-control	−0.053	(−0.064 to −0.043)	*<0.001*

BMI, body mass index; PDSS, paediatric daytime sleepiness scale; SEIFA, Socio-economic Indexes for Areas scale (1–10 with 1 being most disadvantaged and 10 being least disadvantaged).

## Data Availability

The data presented in this study is available on request from the Health4Life Team (info@health4life.org.au). The data is not publicly available due to the ongoing clinical trial.
